# Supplementary material for: A neural crest cell isotropic-to-nematic phase transition in the developing mammalian gut
Source: Commun Biol. 2021 Jun 23;4:770. doi: 10.1038/s42003-021-02333-5 (PMC8222382; doi:10.1038/s42003-021-02333-5)
Supplement: Supplementary file 2 — Supplementary Information [file 42003_2021_2333_MOESM2_ESM.pdf]

Supplementary Material to "A neural crest cell isotropic-to-nematic phase transition in the developing mammalian gut"

Nicolas R. Chevalier[1*], Yanis Ammouche[1], Anthony Gomis[1], Lucas Langlois[1], Thomas Guilbert[2], Pierre Bourdoncle[2], Sylvie Dufour[3]

[1]*Laboratoire Matière et Systèmes Complexes, Université de Paris / CNRS UMR 7057, 10 rue Alice Domon et Léonie Duquet, 75013 Paris, France*

[2] *Institut Cochin, INSERM U1016, CNRS UMR 8104, Université de Paris (UMR-S1016), 75014 Paris, France*

[3] *Univ Paris Est Creteil, INSERM, IMRB, F-94010 Creteil, France*

*Correspondence should be addressed to: nicolas.chevalier@univ-paris-diderot.fr

Corresponding Author ORCID: 0000-0002-9713-1511

Supplementary videos

VideoS1: time-lapse of the circumferential re-orientation of neural-crest derived cells (genetically labels with YFP) in the E14.5 mouse gut cultured for 2 days.

VideoS2: confocal stack of E14.5 duodenum, red: Tomato+ ENCCs, green: collagen I fibers (SHG)

VideoS3: confocal stack of E17.5 duodenum, red: Tomato+ ENCCs, green: collagen I fibers (SHG)

VideoS4: confocal stack of E17.5 hindgut, red: Tomato+ ENCCs, green: collagen I fibers (SHG)

VideoS5: confocal stack of E17.5 duodenum, red: Tuj positive cells (neurons), green: collagen I (whole mount immunohistochemistry and spinning disk confocal microscope).

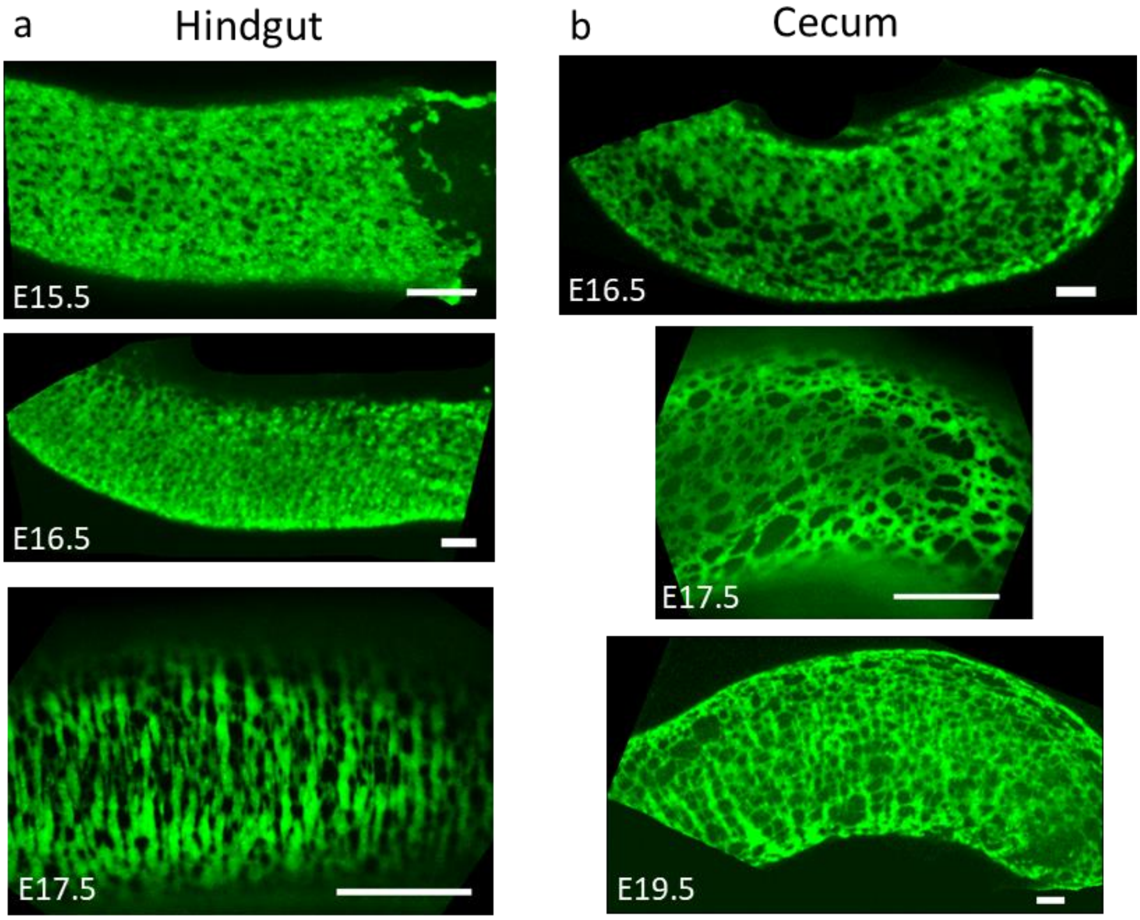

Figure S1. ***Ganglion circumferential orientation dynamics in the hindgut (a) and cecum (b).*** *YFP reporter expression in the cytoplasm of ENCCs**.** Circumferenital orientation of the ENS is visible in the hindgut already at E16.5, and conspicuous at E17.5. In the ceum, we observed orientation in the full cecum only as from E19.5. White bar length = 100 µm.*

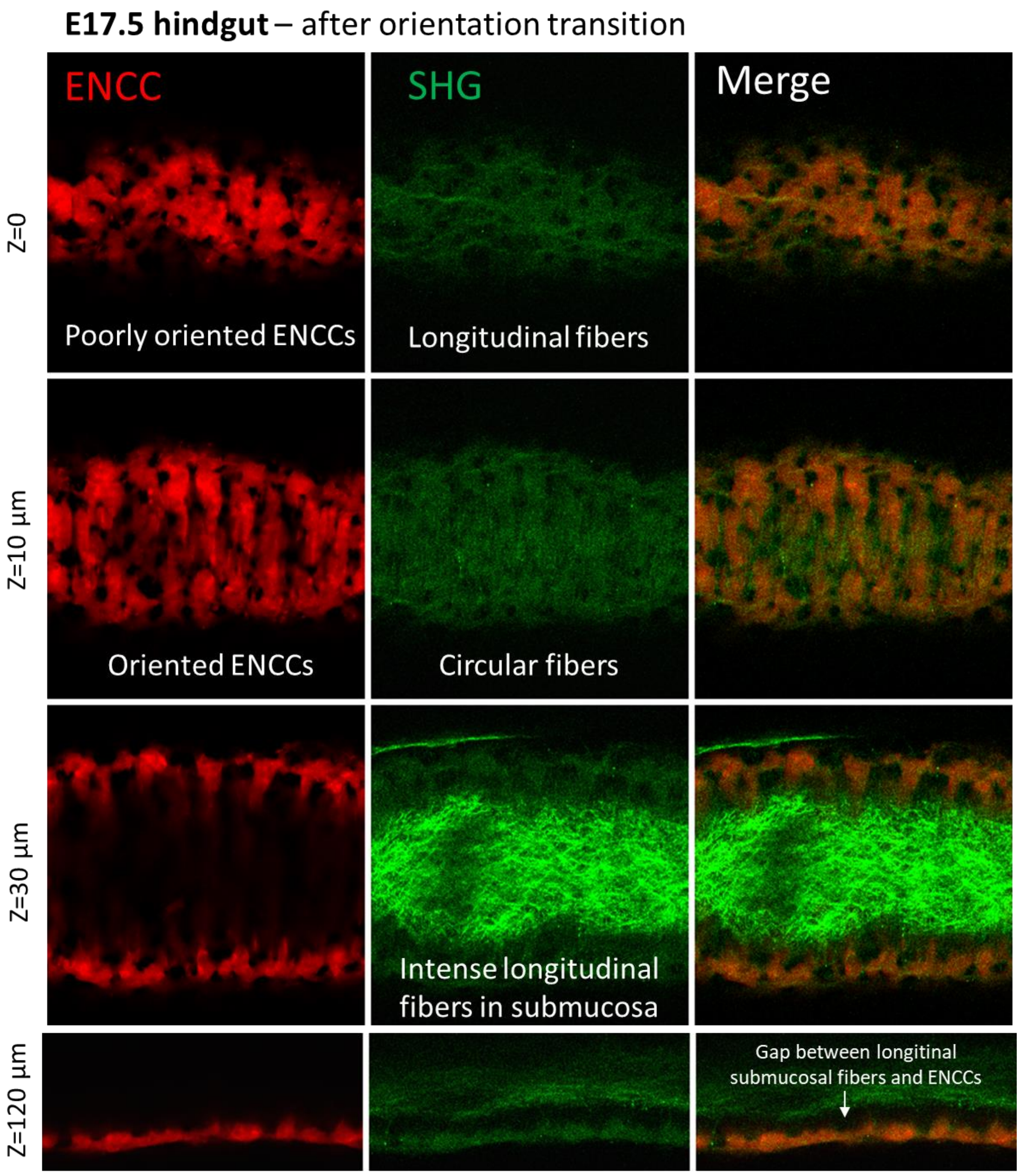

Figure S2. ***ENCC & collagen imaging by second harmonic generation microscopy in the E17.5 hindgut.*** *In the outer layer of the MP (z=0 µm), ENCCs show a low degree of orientation and longitudinal collagen fibers are visible (arrowhead). Deeper inside the MP (z=10 µm), ENCCs and collagen fibers are circularly oriented (arrowhead). Deeper yet inside the gut (z=20 µm), in the submucosa, lies a thick layer of longitudinally oriented collagen fibers (arrowhead) (j) Longitudinal optical section shows that the ENCCs are not in contact with this deeper longitudinal collagen layer, a ~10-20 µm gap separates the two (arrowhead).*

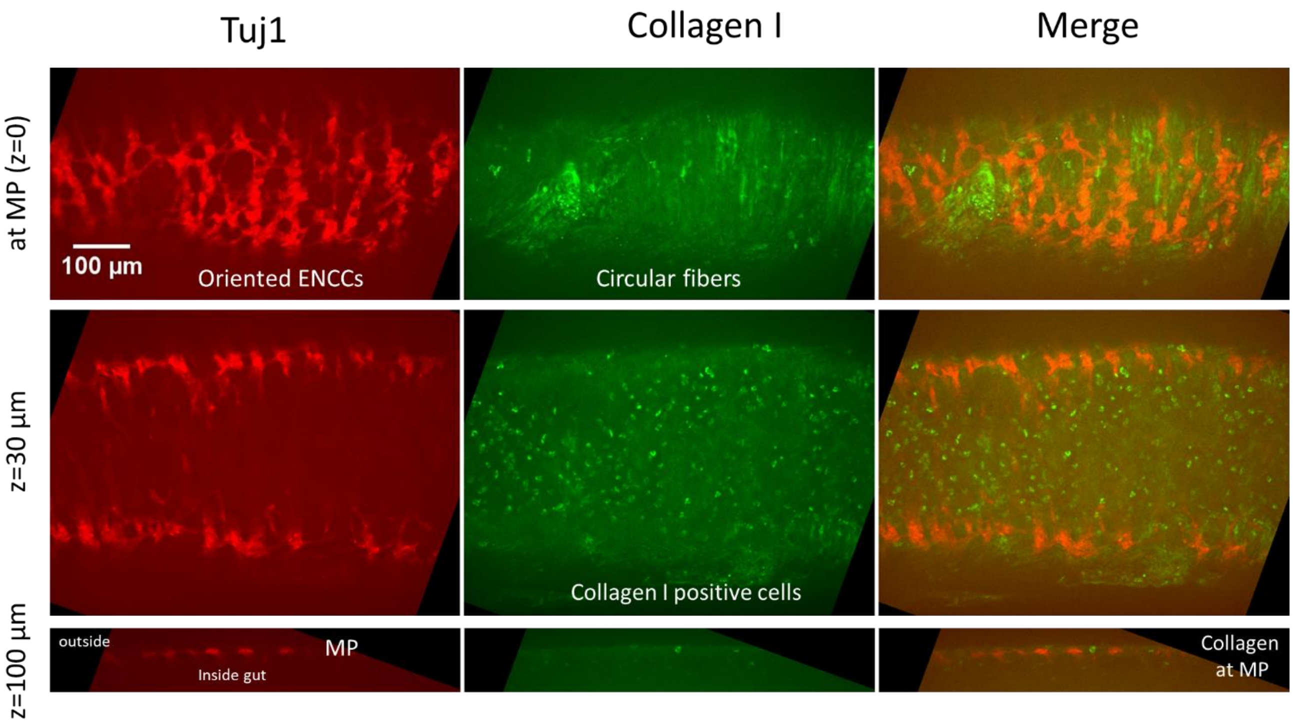

Figure S3. ***Collagen I and Tuj whole mount immunohistochemistry in E17.5 duodenum.*** *Tuj (anti-βIII tubulin) reveals neurons, collagen I was stained by classical whole mount IHC (see Material and Methods) and the gut was imaged with a spinning disk confocal microscope, instead of SHG.*
