# Supplementary material for: A neural crest cell isotropic-to-nematic phase transition in the developing mammalian gut
Source: Commun Biol. 2021 Jun 23;4:770. doi: 10.1038/s42003-021-02333-5 (PMC8222382; doi:10.1038/s42003-021-02333-5)
Supplement: Supplementary file 3 — Description of Additional Supplementary Files [file 42003_2021_2333_MOESM3_ESM.pdf]

Description of Additional Supplementary Files

File Name: Supplementary Movie 1

Description: time-lapse of the circumferential re-orientation of neural-crest derived cells (genetically labels with YFP) in the E14.5 mouse gut cultured for 2 days.

File Name: Supplementary Movie 2

Description: confocal stack of E14.5 duodenum, red: Tomato+ ENCCs, green: collagen I fibers (SHG).

File Name: Supplementary Movie 3

Description: confocal stack of E17.5 duodenum, red: Tomato+ ENCCs, green: collagen I fibers (SHG).

File Name: Supplementary Movie 4

Description: confocal stack of E17.5 hindgut, red: Tomato+ ENCCs, green: collagen I fibers (SHG).

File Name: Supplementary Movie 5

Description: confocal stack of E17.5 duodenum, red: Tuj positive cells (neurons), green: collagen I (whole mount immunohistochemistry and spinning disk confocal microscope).

File name: Supplementary Data 1

Description: Source data underlying the graphs.
